# Comprehensive Genomic and Transcriptomic Analysis of Three Synchronous Primary Tumours and a Recurrence from a Head and Neck Cancer Patient

**DOI:** 10.3390/ijms22147583

**Published:** 2021-07-15

**Authors:** Luisa Bresadola, David Weber, Christoph Ritzel, Martin Löwer, Valesca Bukur, Özlem Akilli-Öztürk, Julia Becker, Hisham Mehanna, Barbara Schrörs, Fulvia Vascotto, Ugur Sahin, Anthony Kong

**Affiliations:** 1 TRON-Translational Oncology, University Medical Center, Johannes Gutenberg University Mainz, Freiligrathstraße 12, 55131 Mainz, Germany; Luisa.Bresadola@TrOn-Mainz.DE (L.B.); David.Weber@TrOn-Mainz.DE (D.W.); Martin.Loewer@TrOn-Mainz.DE (M.L.); Valesca.Bukur@TrOn-Mainz.DE (V.B.); Oezlem.Akilli-Oeztuerk@TrOn-Mainz.DE (Ö.A.-Ö.); j.becker.1998@web.de (J.B.); Barbara.Schroers@TrOn-Mainz.DE (B.S.); Fulvia.Vascotto@TrOn-Mainz.DE (F.V.); 2 University Medical Center, Johannes Gutenberg University Mainz, Langenbeckstraße 1, 55131 Mainz, Germany; Christoph.Ritzel@TrOn-Mainz.DE; 3 Institute of Head and Neck Studies (InHANSE), University of Birmingham, Vincent Drive, Birmingham B15 2TT, UK; H.Mehanna@bham.ac.uk; 4 HI-TRON, Helmholtz Institute for Translational Oncology Mainz—A Helmholtz Institute of the DKFZ, Freiligrathstraße 12, 55131 Mainz, Germany; 5 Comprehensive Cancer Centre, Guy’s Campus, King’s College London, Room 2.36b New Hunt’s House, Newcomen St, London SE1 1UL, UK

**Keywords:** synchronous multiple primary malignancies, recurrent head and neck squamous cell carcinoma (HNSCC), somatic single nucleotide variants, germline variants, immune cell infiltration, whole exome sequencing (WES), RNA sequencing (RNA-seq)

## Abstract

Synchronous primary malignancies occur in a small proportion of head and neck squamous cell carcinoma (HNSCC) patients. Here, we analysed three synchronous primaries and a recurrence from one patient by comparing the genomic and transcriptomic profiles among the tumour samples and determining the recurrence origin. We found remarkable levels of heterogeneity among the primary tumours, and through the patterns of shared mutations, we traced the origin of the recurrence. Interestingly, the patient carried germline variants that might have predisposed him to carcinogenesis, together with a history of alcohol and tobacco consumption. The mutational signature analysis confirmed the impact of alcohol exposure, with Signature 16 present in all tumour samples. Characterisation of immune cell infiltration highlighted an immunosuppressive environment in all samples, which exceeded the potential activity of T cells. Studies such as the one described here have important clinical value and contribute to personalised treatment decisions for patients with synchronous primaries and matched recurrences.

## 1. Introduction

Head and neck squamous cell carcinomas (HNSCCs) are associated with smoking and alcohol intake, with human papillomavirus (HPV) infection accounting for the increasing incidence of oropharyngeal cancer [1]. Patients with HNSCC are also at elevated risk of second primary malignancies. *Metachronous* second primary malignancies (occurring more than six months after diagnosis of the index HNSCC) occur in 20% to 30% of HNSCC patients [2,3,4]. This is most likely due to field change cancerisation from long-term tobacco or alcohol exposure, which may induce mucosal changes associated with premalignant disease, as well as increased cancer frequency in the mucosal sites of the head and neck, lung, and oesophagus [2,3,4]. Patients with index HPV-associated cancers (such as cervix or anal SCC) also have a significantly higher risk of oropharyngeal HPV-associated second primary cancers [5]. *Synchronous* primary malignancies (SPMs; occurring within six months from the diagnosis of the first primary cancer) are instead present in a smaller proportion of patients, around 1% to 6% of patients with newly diagnosed HNSCC [2,3,4]. The risk of developing SPMs varies depending on the index HNSCC subsite: among the HNSCC subsites, oropharyngeal cancers carry the lowest risk of SPMs compared to non-oropharyngeal SCC, despite a rising incidence of oropharyngeal SCC due to HPV infection [5,6]. In the presence of multiple synchronous primary tumours, treatment plans and the sequence of appropriate therapies for each primary tumour may need to be modified, especially if they occur at different sites (e.g., oral cavity and lung) [5,6].

Here, we report a case of a male patient with three synchronous primary HNSCCs, who underwent resection of all three cancers without adjuvant postoperative radiotherapy, but subsequently presented with local and distant recurrences. We performed a comprehensive whole-exome and RNA sequencing experiment of the three primary tumours and the local recurrence: with this, we assessed the heterogeneity among the primary tumours and ascertained the origin of the recurrence, as well as determined the presence of germline variants that could have predisposed the patient to develop multiple malignancies.

### Case Description

**Figure 1:**
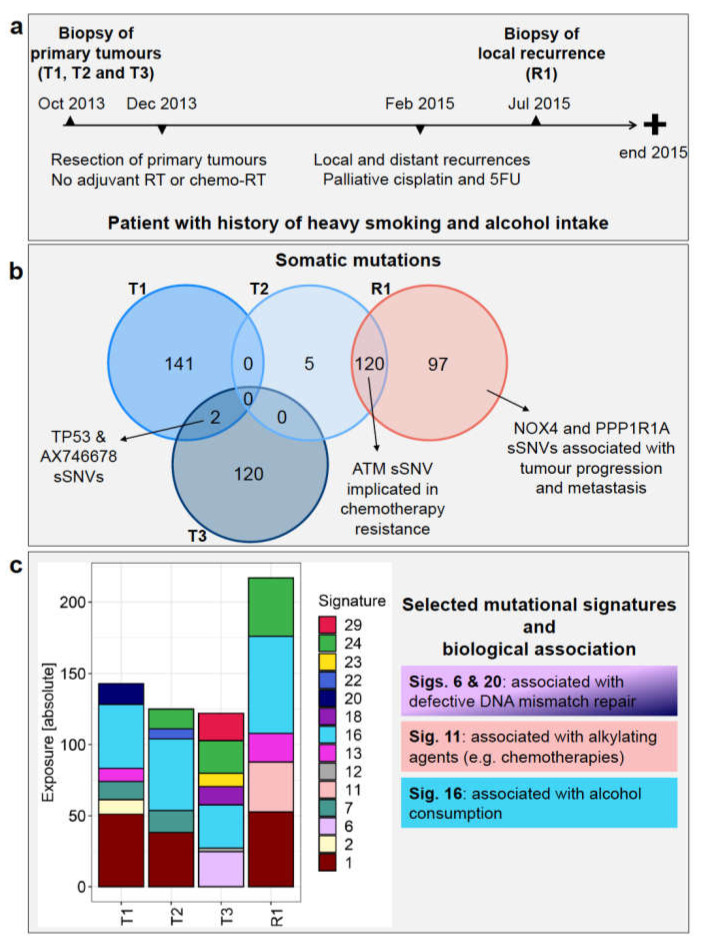
Patient history and genomic landscape of somatic mutations. (**a**) Patient’s timeline of diagnosis, treatment, and recurrence. (**b**) The Venn diagram shows the number of somatic single nucleotide variants (sSNVs—both synonymous and non-synonymous) identified from WES data in each of primary tumours T1, T2, T3, and recurrent tumour R1 and any shared mutations (left). (**c**) Absolute exposure of the mutational signatures detected in T1, T2, T3, and R1 is shown, along with selected biological associations. *RT* = radiotherapy, *5FU* = fluorouracil.

The patient was a male with a long history of heavy smoking and high alcohol intake (Figure 1a). He presented with three synchronous primary tumours in October 2013 at the age of 68 and underwent three biopsies: primary tumour 1 (referred here as T1) from the right soft palate, primary tumour 2 (T2) from the left tongue, and primary tumour 3 (T3) from the posterior pharyngeal wall. Clinically, the three primary tumours were SCCs in three separate locations, and all were p16 negative, as determined by immunohistochemistry (IHC) carried out as part of the standard of care. The patient underwent a resection of all three primary tumours in December 2013. The resected sample from the right soft palate (T1) showed squamous epithelial dysplasia but several islands of moderately differentiated SCC towards the posterior pole, stage pT1N0M0 (pR1 resection). The resected sample from the left tongue hemiglossectomy with neck dissection (T2) showed a large ulcero-infiltrative invasive moderately/poorly differentiated SCC, stage pT3N2cM0 (pR0 resection). The resected sample from the posterior pharyngeal wall (T3) showed central severe dysplastic surface squamous epithelium with islands of early invasive, well-differentiated SCC pT1N0M0 (pR0 resection). The patient did not receive any adjuvant postoperative radiotherapy or chemoradiation. He subsequently presented in February 2015 with a large recurrence of the left posterior tongue abutting mandible (referred here as R1) and involving the lateral pharyngeal wall encircling external carotid artery with multiple left neck lymphadenopathy and mediastinal/pleural nodules. He underwent a course of palliative cisplatin and fluorouracil (5FU) chemotherapy but progressed. A biopsy of the recurrent tumour R1 (also p16 negative) was taken in July 2015 while the patient was having chemotherapy before the patient succumbed to the disease and died in 2015.

## 2. Methods

### 2.1. Patient Samples

Biopsies and resected samples from the three primary tumours and the recurrence, together with the patient’s buffy coat, were obtained from the University of Birmingham Biobank (patient previously consented through Accelerated programme, Research Ethics Committee West Midlands ref. 14/YH/1101). The tumour samples were conserved as formalin-fixed paraffin-embedded (FFPE) blocks. The resected tumour samples were subsequently not used for next-generation sequencing analysis due to low tumour content in T1 and T3. All the samples used for analysis here were from biopsy samples. R1 was collected while the patient was receiving palliative chemotherapy, as indicated in the Case description above.

### 2.2. Next-Generation Sequencing Data

Sections of the FFPE blocks were subjected to DNA extraction using the Maxwell FFPE DNA kit and following the manufacturer’s instructions. Matched normal DNA was extracted from the buffy coat of the patient using the Qiagen Blood and Tissue Kit. Exome capture libraries were prepared in duplicates from the DNA of the tumour and normal DNA samples using the SureSelect Human All Exon V6 kit (Agilent) and sequenced paired-end 2 × 50 nt on an Illumina NovaSeq 6000. RNA was extracted from the tumour samples with the FFPE Clear Kit from AmpTech. Using 130 to 814 ng input RNA, libraries were prepared with the Illumina TruSeq RNA Library Prep Kit V2 and sequenced paired-end 2 × 50 nt on an Illumina HiSeq 2500.

### 2.3. Bioinformatic Data Processing

*Exome-seq data*. The whole-exome sequencing (WES) reads from tumour and normal samples were aligned to the human reference genome hg19 with bwa 0.7.10 [7], and duplicate reads were flagged with MarkDuplicates from the Picard Tools suite 1.110 (http://broadinstitute.github.io/picard/, accessed on accessed 12/02/2019). Somatic single nucleotide variants (sSNVs—also called here “somatic mutations”) were identified with an established mutation detection process [8], which compares the sequencing data from the tumour and the normal sample of the patient. In addition to a list of sSNVs, the programme also generates a list of candidate mutation sites and an estimate of the purity of the tumour samples. Replicate libraries are incorporated explicitly into the mutation calling process by testing both the sum of replicates and replicates separately for the existence of a somatic mutation and computing a combined result. The genomic positions of the somatic mutations were overlapped with the UCSC known genes transcript coordinates in order to associate the sSNVs with genes and potential amino acid sequence changes.

The overlap of sSNVs among tumour samples was then evaluated in R 3.5.1 [9] to quantify the fraction of somatic mutations unique to each primary sample, to the recurrence or shared between these. To avoid false negatives, the sSNVs detected in each tumour sample were grouped, and for each of these, we verified its presence in the list of candidate mutation sites of the remaining tumour samples. If the sSNV was also not present in this list, we proceeded to inspect the sequencing reads aligning at the mutation position: if the depth was ≥50 and the variant allele frequency (VAF) was < 0.01, the sSNV was considered absent in the specific tumour sample; if the depth was ≥ 50 and VAF was > 0.02, the sSNV was considered present, while if these conditions were not satisfied we excluded the sSNV from the analysis.

Based on the final sSNV lists, COSMIC mutational signatures (v2—March 2015) were calculated for each tumour sample using the R package YAPSA [10]. The WES data were analysed with Control-FREEC v11.5 [11] to obtain copy number (CN) information from the tumour samples. We used the parameter values recommended for this type of sequencing data and provided a genome mappability file (downloaded from https://xfer.curie.fr/get/fdwcA3QfU5a/GEM_mapp_hg19.tar.gz (accessed on 10 March 2021)) and the tumour content estimated via histological inspection (Table 1) as additional parameters. Consistency in the results obtained from the two WES replicates and similarity among CN profiles of different tumour samples was evaluated by calculating Spearman’s correlation coefficients between the median ratios (tumour/normal) output by the program.

Germline variants were called from the normal sample with DeepVariant 0.9.0 [12], comparing the patient’s sequence to the human reference genome hg19. The vcf files obtained from the replicate WES libraries were filtered separately with custom scripts and bcftools 1.9 [13], retaining only the variants detected in exons, with FILTER information equal to “PASS” and covered by at least eight reads. Mutations with coverage higher than the 99% quantile of the depth distribution were removed to exclude potentially paralogous sites, as were also those with a QUAL value lower than 20. The vcf files were then merged with bcftools 1.9 (merge command) [13]. Multi-allelic sites were filtered out, while variants found only in one of the two replicates were retained. The final set of mutations was annotated with SnpEff 4.3t [14] using only canonical transcripts and ANNOVAR 2018Apr16 [15]. To focus on a smaller set of relevant mutations, we retained only those affecting genes included in the cancer gene list from OncoKB [16] (downloaded on 27 September 2019) and having “moderate” or “high” impact, according to SnpEff. We further excluded the mutations with a minor allele frequency ≥ 0.05 in the Non-Finnish European population, according to the WES data in gnomAD [17]. When the allele frequency from WES data was not available, we used the value derived from genome data in gnomAD.

*RNA-seq* data. RNA-seq reads were aligned with STAR 2.4.2a [18] to the human reference genome hg19. Expression analysis was performed with custom Python scripts, which intersect the UCSC exon coordinates with the read alignments, count the number of reads, and normalize to RPKM (reads per kilobase of transcript, per million mapped reads [19]).

Genes overexpressed in the tumour samples were identified by comparing expression values to those calculated in a cohort of 42 unrelated HNSCC adjacent normal tissue samples from TCGA, which were analysed with the same expression pipeline used for the in-house data. A gene was considered overexpressed when its expression was above 10 RPKM and at least 10-fold higher than the median expression of the TCGA adjacent normal samples. Subsequent Gene Ontology (GO) analysis was performed with the Database for Annotation, Visualization and Integrated Discovery (DAVID) v6.8 [20,21] to identify significantly enriched GO terms (Benjamini–Hochberg adjusted *p*-value < 0.05).

RNA-seq reads were also used as input for quanTIseq, which quantifies the absolute fractions of immune cells in tumour samples through a novel and validated deconvolution approach [22]. Results from quanTIseq were used to calculate the ratio between M1 and M2 macrophages, as well as the percentage of immunosuppressive cell subsets. The latter was obtained by adding the fractions of regulatory T cells and M2 macrophages, dividing this number by the total fraction of immune cells in the sample and multiplying times 100 to obtain a percentage.

### 2.4. Histology and Immunohistochemistry Assays

Three-μm FFPE tissue sections were stained with haematoxylin and eosin (H&E). Briefly, after deparaffinisation and rehydration, sections were incubated in Hemelaum (acc. to Mayer) solution for five minutes and counterstained with eosin for two minutes. Consecutive sections were stained in the Leica BOND Rx autostainer using a mouse anti-human CD3 antibody (ready to use, Leica Bioscience, PA0553-CN) and a rabbit anti-human PD-L1 antibody (1:400, Cell Signaling, 13684S). The stainings were detected using BOND Polymer Refined Detection Kit (Leica, DS9800). Manual multiplexed IHC staining was performed using tyramide-conjugated Opal fluorophores. After heat-induced epitope retrieval at 95 °C for 30 min and blocking, the primary antibody rabbit anti-human CD163 (1:500, Cell Signalling, 93498S) was incubated on slides at 4 °C overnight, followed with horseradish peroxidase (HRP)-conjugated secondary antibody (ready to use, Immunologic, DPVR-110HRP). The target was then detected with the tyramide-conjugated fluorophore (Opal 570, 1:250, Akoya Bioscience, FP1488A). A heating step was introduced to deactivate the primary and secondary antibody complex before incubating the slides with the primary antibody rabbit anti-human CD68 (1:2000, Cell Signalling, 76437S) for 1 h at room temperature. The second target was detected with the tyramide-conjugated fluorophore (Opal 520, 1:250, Akoya Bioscience, FP1487A) after incubation with a secondary antibody. A heating step was introduced again to deactivate the primary and secondary antibody complex, followed by counterstaining with DAPI (Akoya Bioscience, FP1490). The microscope images were taken using the Zeiss Axioscanner and Akoya Bioscience Vectra Polaris scanner microscopes. The examination of the tissue for tumour content and immune cell infiltration was performed by a board-certified pathologist.

## 3. Results

### 3.1. Next-Generation Sequencing Read Number and Depth

We performed WES of the primary tumours, the recurrent tumour, and the matched normal sample. The tumour samples yielded an average number of 236.77 million read pairs across replicates and samples (sd 24.10 million), while for the normal sample, the average was 246.14 million read pairs across replicates (sd 15.61 million). For the tumour samples, the mean target depth was 295.72 (sd 35.92) with an average fold enrichment of 40.42 (sd 2.10), whereas, for the normal sample, the mean target depth was 277.81 (sd 17.05) with an average fold enrichment of 35.77 (sd 0.08).

In addition, we performed transcriptome sequencing (RNA-seq) of the tumour samples and obtained an average of 35.10 million read pairs (sd 4.06 million), with an alignment rate to hg19 of 96.94% (sd 0.006) and 65.92% (sd 11.42) of the reads aligning to transcripts.

### 3.2. Somatic Mutations Reveal High Degree of Heterogeneity among Primary Tumours and the Origin of the Recurrence

The total number of somatic single nucleotide variants (sSNVs, and the number of non-synonymous sSNVs), the tumour purity calculated by the mutation calling pipeline, the percentage of tumour area and nuclei deriving from the histological inspection of primary and recurrent tumours are reported in Table 1.

The sets of somatic mutations identified in the WES data varied significantly among the three primary tumours. Only two sSNVs were shared between T1 and T3, namely a non-synonymous mutation found in TP53 (Y181C) and a synonymous one found in AX746678 (Figure 1b and Supplementary Table S1a). No common sSNVs were detected between T2 and the other two primary tumours, suggesting that these three samples represent SPMs (Figure 1b and Supplementary Table S1a).

R1 affected the left posterior tongue and the lateral pharyngeal wall with multiple left neck lymphadenopathy and pleural nodules, therefore from a clinical point of view, it could have originated from any of the three primary tumours. None of the mutations in the recurrent tumour, however, was found in T1 and T3, whereas 120 mutations were shared between T2 and R1 (Figure 1b and Supplementary Table S1a). This observation strongly infers that the recurrent tumour originated from T2. The mutation patterns also fit with the clinical picture since T2 presented with the most advanced disease (T3N2cM0) compared to T1 and T3 (both T1N0M0 at presentation).

We evaluated if CN estimates could also contribute to determining the origin of the recurrence. The results were consistent across the WES replicates: correlation coefficients among the median ratios of the two replicates were very high for T2, T3, and R1 (rho = 0.904, 0.900, 0.975, respectively; *p*-value < 2.2 × 10^−16^), while the correlation was lower for T1 (rho = 0.766, *p*-value < 2.2 × 10^−16^; Supplementary Figure S1). As can be observed from the heat map, the CN profile of R1 was most closely related to the one of T2, while T1 and T3 formed separate clusters (Supplementary Figure S1). This result provided additional support for the origin of R1 from primary T2. However, given the uncertainty in CN estimation for sample T1, it is advisable to interpret these results with caution. Somatic mutation patterns provided, therefore, more compelling evidence to ascertain the origin of R1.

**Figure 2:**
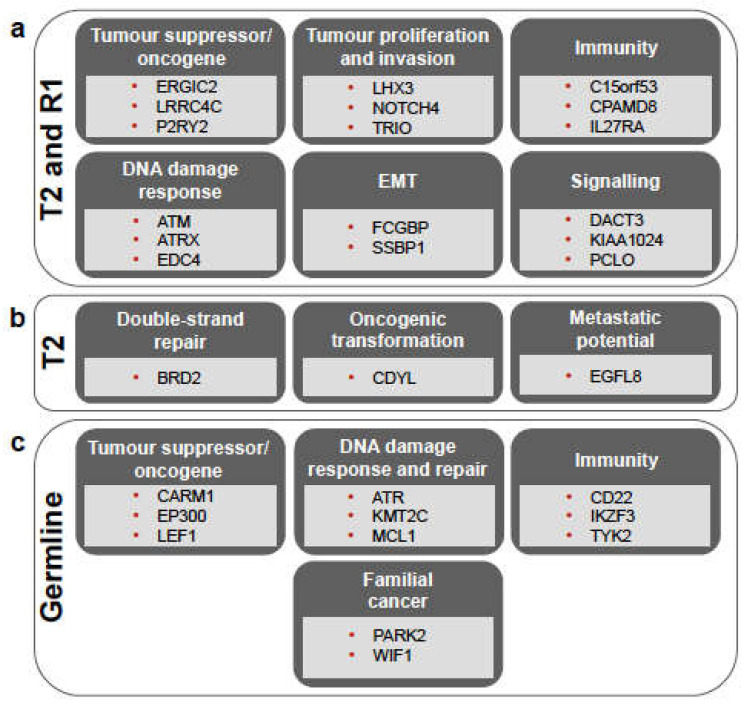
Overview of the main biological processes in which mutated genes are involved, shown for the somatic mutations shared between T2 and R1 (**a**), those unique to T2 (**b**), and for the germline variants (**c**). For more details, see Supplementary Tables S1b, S1c, and S4b, respectively. *EMT* = epithelial-mesenchymal transition.

Among the sSNVs shared by T2 and R1, 75 were non-synonymous (Supplementary Table S1a), and many of these were found in genes with either oncogenic or tumour suppressor function in various cancers (Figure 2a and Supplementary Table S1b). For the genes with known roles in tumour suppression (e.g., LRRC4C, ERGIC2, GJA9, PHLPP2, and SERPINI2) or oncogenic function if upregulated (e.g., KLC2, P2RY2, ESPL1, INF2, ARHGAP33, ENPP3, and FAM166B), we report the gene expression values of the three primary tumours and recurrent tumour (Supplementary Table S2a), but we did not observe evident differences between them. Mutations shared between T2 and R1 were also found in DNA damage response genes, such as ATM (H2887L), EDC4 (H966Q—known to interact with BRCA1 [23]), and ATRX (Y1847C; Figure 2a and Supplementary Table S1b). In these cases as well, no obvious difference in gene expression could be observed in the three primary tumours and the recurrent tumour (Supplementary Table S2a). The list of mutations shared between T2 and R1 also included non-synonymous sSNVs detected in genes involved in innate and adaptive immunity, such as C15orf53, CPAMD8, ZCCHC11, and IL27RA (Figure 2a and Supplementary Table S1b).

Five mutations were unique to T2, not being detected in R1, T1, or T3 (Figure 1b). Three of these were non-synonymous mutations, namely those affecting EGFL8 and the read-through PPT2-EGFL8 (L288F), BRD2 (R511Q), and CDYL (E459V), while the other two (detected in FNDC4 and FREM1) were synonymous mutations (Supplementary Tables S1a and S1c). Both EGFL8 and CDYL genes were described as tumour suppressors, while BRD2 is involved in DNA double-strand repair, interacts with RUNX3, and affects p53 stability, thus influencing their efficiency as tumour suppressors (Figure 2b and Supplementary Table S1c). There was, however, no clear difference in the expression of these genes among the four tumour samples (Supplementary Table S2a).

Furthermore, 97 mutations (of which 72 are non-synonymous) were detected in R1 but not in T2 (Figure 1b and Supplementary Table S1a). Many of the mutated genes, such as NOX4 [24] and PPP1R1A [25], have been implicated in tumour progression and/or metastasis.

Regarding the sSNVs detected only in the other primaries, 141 were found in T1 alone (Figure 1b; Supplementary Table S1a), affecting genes such as PRDM9 and TET2, which play a role in DNA repair [26,27], as well as VEGFB, MLL, STAT3, and STAT4, which could play key roles in immune regulation [28,29,30,31]. In addition, T3 had 120 mutations not found in the other tumours (Figure 1b; Supplementary Table S1a), including, for example, variants in ILF2 and IL7R, which regulate immune function [32,33], and in EP400 and POU2F1, which contribute to DNA repair mechanisms [34,35].

### 3.3. Germline Variants Indicate Cancer Predisposition in the Patient

We analysed the germline variants of the patient to investigate his genetic predisposition to develop SPMs. In the replicate WES libraries of the normal sample, DeepVariant identified 571,907 and 465,016 potential variants, respectively. After the filtering steps, we retained 34,026 variants, which were annotated with SnpEff and ANNOVAR (see Methods section). Of these, 1837 germline variants were found in the OncoKB cancer genes, and 449 were annotated as having “moderate” or “high” impact (Supplementary Table S3). BRCA1 and BRCA2, as well as PTEN variants, were found in this list (Supplementary Table S3), but their frequency in the Non-Finnish European population is ≥0.05, and they are thus likely benign, as also suggested by the ClinVar annotation. These variants, together with the others frequently occurring in the non-Finnish European population, were therefore filtered out to obtain a final set of 41 germline variants (Supplementary Table S4a). Of these, several are found in genes involved in DNA damage response and double-strand DNA repair mechanisms, including MCL1, ATR, KMT2C, and CBLC, indicating genomic instability and cancer predisposition (Supplementary Table S4b). A few genes affected by germline variants were previously found to be involved in familial cancers, such as PARK2 [36] and WIF1 [37], while others are known to be tumour suppressor genes involved in the p53 pathway, including LEF1, NUMA1, BUB1B/BUBR1, CARM1, EP300, GTSE1 and MYH9 (Supplementary Table S4b). In addition, we also found germline variants in genes regulating immune response and immune surveillance, including IKZF3, TYK2, and CD22 (Supplementary Table S4b).

### 3.4. Mutational Signature Analysis Reflects Diverse Mechanisms Involved in Both Tumorigenesis and Patient History

In the sSNVs detected in R1, we identified mutational signatures 1, 11, 13, 16, and 24, among which signatures 1, 13, and 16 were shared with T1, signatures 1, 16, and 24 with T2 and signatures 16 and 24 with T3 (Figure 1c).

Signature 1 is the result of an endogenous mutational process, whereas Signature 24 is related to guanine damage repaired by transcription-coupled nucleotide excision repair [38,39]. Signature 11 was found only in the recurrent tumour and not in any of the primary tumours. This signature exhibits a mutational pattern resembling that of alkylating agents [38,39]. Therefore, its presence in R1 is most likely treatment-related since this patient received cisplatin and 5FU chemotherapy before the biopsy of R1 was performed.

In all tumours, including the recurrence, we detected Signature 16, which is associated with alcohol consumption and has been frequently reported in oesophageal squamous cell carcinoma, liver cancer, and HNSCC [23], consistent with the patient being a heavy alcohol drinker. Li and colleagues [40] reported an increased mutational activity of Signature 16 associated with mutations in the genes ZNF750, TP53, and EP300. The association between TP53 mutation status and Signature 16 was highlighted by a significantly higher fraction of TP53 T>C mutations in the alcohol group versus the non-alcohol group [40]. In this patient, a TP53 T>C mutation (Y181C) was found in primary tumours T1 and T3 (Supplementary Table S1a). In addition, germline variants in EP300 and in other genes affecting p53 function (e.g., LEF1, NUP98, NUMA1, BUB1B/BUBR1, CARM1, GTSE1, and MYH9) were also detected (Supplementary Table S4b). This could explain the presence of Signature 16 in all the primary and recurrent tumours.

Signature 13 was found in T1 and R1 (Figure 1c). This signature has been found in 22 cancer types and has been attributed to the activity of the AID/APOBEC family of cytidine deaminases, which convert cytosine to uracil [38].

Although T2 gave rise to R1, loss of Signatures 7 and 22 occurred between T2 and R1 (Figure 1c): Signature 7 was previously found in head and neck oral SCC [38], while Signature 22 was found in cancer samples with known exposures to aristolochic acid, including liver cancer and urothelial carcinoma [39]. Mutational signatures are calculated by taking into account the whole spectrum of mutations present in a sample, and those that best explain the observed patterns are reported. Since R1 had a higher mutational burden than T2, it is not surprising that Signatures 7 and 22 were not present in R1, in spite of the remarkable proportion of shared sSNVs between these samples. Among the four mutational signatures (6, 15, 20, and 26) associated with defective DNA mismatch repair [38], two (20 and 6) were found in primary tumours T1 and T3, respectively, consistent with DNA repair gene mutations, namely PRDM9 and TET2 [24,25] in T1, and EP400 and POU2F1 [32,33] in T3, as well as potential genetic predisposition, as described above.

### 3.5. Heterogeneity across Tumour Samples Is Observed in Gene Expression Patterns

**Figure 3:**
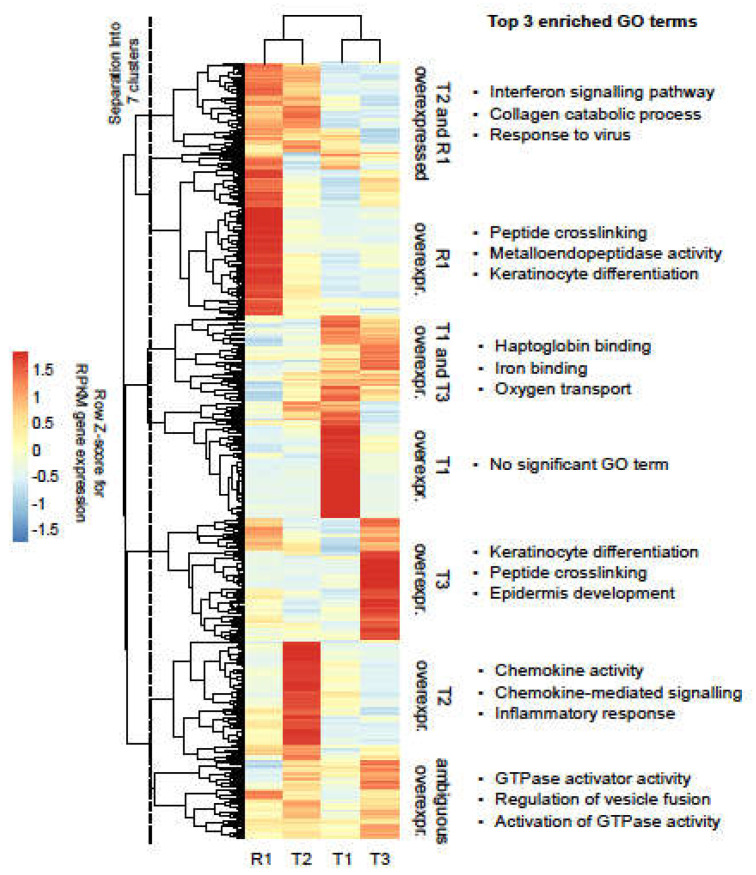
Overexpressed genes in the tumour samples. Heat map representation of tumour overexpressed genes. RPKM expression values are represented as row Z-scores to highlight differences. Unsupervised clustering (complete linkage with Euclidean distances) was applied to samples and genes. Following visual inspection, genes were separated into seven major clusters with sample-specific expression patterns, as indicated by the vertical dashed line.

Gene expression in the tumour samples was compared to the expression of a cohort of unrelated HNSCC adjacent normal tissue samples in order to identify tumour overexpressed genes. We identified 631 overexpressed genes, which showed noticeable heterogeneity in the expression levels across the tumour samples. After visual inspection of the results, overexpressed genes were thus divided into seven clusters, according to their expression patterns (Figure 3 and Supplementary Table S2b). Applying unsupervised clustering, the recurrence R1 was found to be most closely related to primary tumour T2, also in terms of gene expression.

In the clusters corresponding to genes overexpressed in T3 and in R1, the GO term analysis revealed significant enrichment for genes related to the differentiation of keratinocytes, consistent with the patient’s tumours being squamous cell carcinoma. Of note, genes overexpressed in R1 were significantly enriched for metalloendopeptidases (MMPs), known to promote cancer progression and metastasis (Figure 3).

### 3.6. Immune Infiltration in the Tumours Correlates with an Immunosuppressive Environment

**Figure 4:**
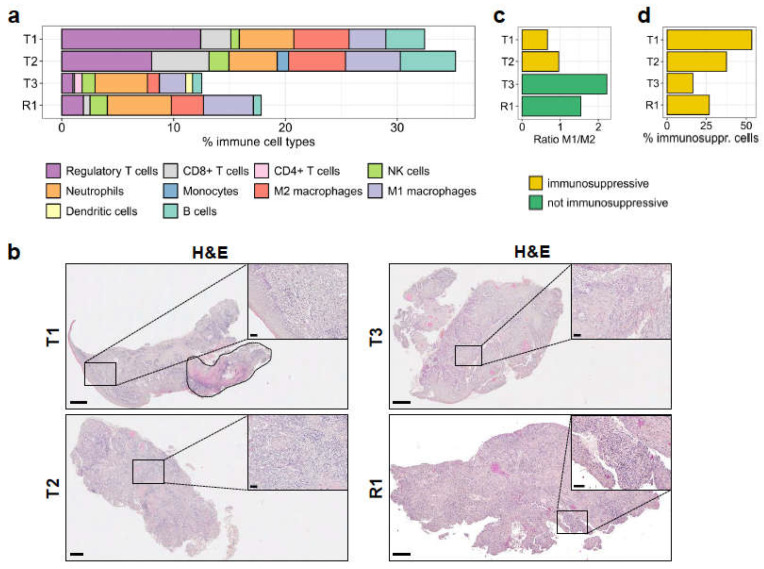
Immune cell profile of the tumour samples. (**a**) The absolute fractions of immune cell types infiltrated in the primary and in the recurrent tumours, as determined by quanTIseq from RNA-seq reads, are shown. (**b**) Histological analysis by H&E staining. Areas with high immune cell infiltration in the tumour are shown in the insets. T1 is a mixture of squamous cell carcinoma and carcinoma in situ, which is marked by a dashed line (top left). Scale bars: 500 μm for full-size images and 50 μm for insets. (**c**) The ratio of M1/M2 macrophage fractions in the samples, derived from quanTIseq deconvolution. (**d**) Immunosuppressive cells (regulatory T cells, M2 macrophages) as a percentage of the total fraction of infiltrated immune cells.

As stated above, R1 originated from one specific primary tumour (T2), suggesting that mechanisms of immunological escape occurred in this particular tumour, favouring relapse. Therefore, we wanted to characterise the immune cells present in the tumour samples, and estimate the proportion of the major immune cell subsets from the RNA-seq data (Figure 4a). Using the quanTIseq pipeline [22], we found a higher level of immune infiltration in T1 and T2 (32.4% and 35.2% of the samples were represented by immune cells) compared to T3 and R1 (12.5% and 17.8%, respectively). Histological analysis of tumour tissues by H&E staining (Figure 4b) confirmed the RNA-seq data: all samples showed strong immune cell infiltration into the tumour (Figure 4b). Immune cells are easily recognisable by their highly dense basophilic nuclear staining and by their small cytoplasmic rim in H&E stainings (insets in Figure 4b). As further validation of the quanTIseq results, we performed an IHC staining to detect the presence of T cells in the tumours. T1 and T2 were highly infiltrated by T cells in the tumour and the tumour stroma (Supplementary Figure S2). In contrast, T3 displayed significantly fewer T cells, which were also exclusively located in the tumour stroma (Supplementary Figure S2, top right inset). A lower fraction of T cells were also detected in R1; however, in this sample, there were fewer T cells infiltrating into the tumour (Supplementary Figure S2, bottom right inset). Although the IHC staining was performed on a different tissue section from the tumour sample used for RNA-seq analysis, the IHC images and the quanTIseq results correlated very well in regards to the fractions of T cells in the tumours.

The quanTIseq analysis also showed high similarity in the type and frequency of immune cell populations infiltrated in T1 and T2 samples. Although these tumours displayed high infiltration of CD8+ T cells, NK, and B cells (Figure 4a), thus representing potentially hot tumours, overall, they showed a strong immunosuppressive environment with a very high frequency of regulatory T cells, as well as of M2 macrophages. The very low M1/M2 ratios (0.66 and 0.96, respectively; Figure 4c) implied a strong polarisation of macrophages to the immunosuppressive M2 phenotype. The macrophage polarisation was confirmed by multiplexed IHC staining using CD163 marker for M2 polarization of CD68+ macrophages (Supplementary Figure S3). Overall, immunosuppressive cell subsets accounted for 53.5% and 37.3% of the immune infiltrates in T1 and T2, respectively (Figure 4d).

On the contrary, T3 showed opposite immunological features: it displayed remarkably low local immune suppression (16.4%; Figure 4d), considering the low proportion of regulatory T cells (Figure 4a), as well as the very high M1/M2 ratio of macrophages (2.22; Figure 4c and Supplementary Figure S3). In addition, quanTIseq detected CD4+ T cells and dendritic cells only in the T3 sample, together with a preserved proportion of NK cells, all inferring a potential anti-tumoural immune response. However, the very low frequency of CD8+ T cells would not have supported a favourable prognosis.

Interestingly, the R1 sample, described here to originate from T2 and collected after five months of palliative 5FU chemotherapy, displayed a lower frequency of immunosuppressive cell subsets (regulatory T cells and M2 macrophages) among total immune cells than T2 (27%; Figure 4d) and a comparably high frequency of M1 macrophages compared to M2 macrophages (Figure 4c and Supplementary Figure S3), while still carrying a similar fraction of NK cells. In addition, the R1 sample, similarly to T3, showed reduced proportions of CD8+ T cells and B cells (Figure 4a).

Among the subsets of myeloid cells, the proportion of neutrophils, which is often referred to as myeloid-derived suppressor cells (MDSC), did not change among the different samples (Figure 4a), and these cells might have strongly contributed to the local immune suppression, together with regulatory T cells and M2 macrophages. An additional investigation should be conducted to properly characterise the heterogeneity of this immune cell subset.

**Figure 5:**
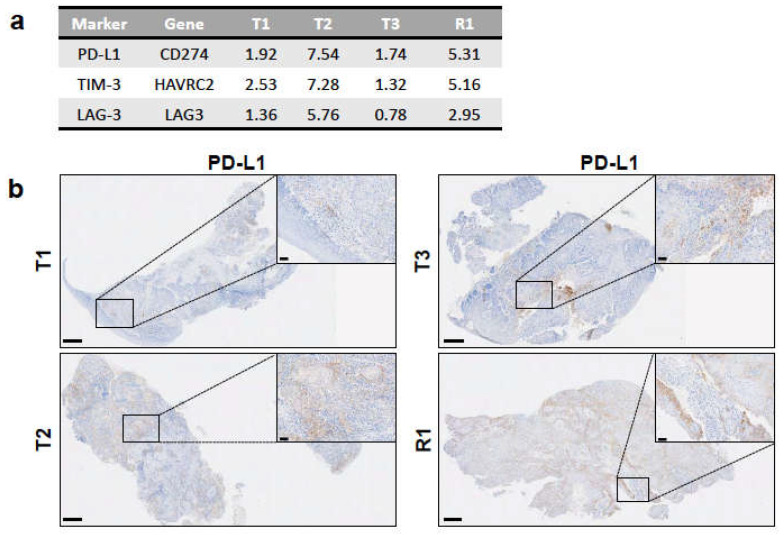
Expression of immunosuppressive markers among tumour samples. (**a**) Expression levels of genes encoding for the immunosuppressive markers PD-L1, TIM-3, and LAG-3 from RNA-seq data as RPKM values. (**b**) PD-L1 expression in each tumour sample as determined by IHC with anti-PD-L1 antibody on FFPE sections. Insets depict positively stained tumor cells in T2 and R1 and immune cells in T1 and T3 (see Results). Scale bars: 500 μm for full-size images and 50 μm for insets.

We also examined the expression levels of specific immunosuppressive molecules in the RNA-seq data of the primary tumours and the recurrence: PD-L1 (encoded by CD274), mainly expressed on tumour and myeloid cells, and TIM-3 (HAVRC2) and LAG-3 (LAG3), both of which are typically expressed on exhausted T cells. All samples had the detectable expression of these three genes, with T2 and R1 having higher values than T1 and T3 (Figure 5a). In agreement, IHC staining showed that PD-L1 expression was detectable in all samples and only highly expressed on the tumour cells in T2 and R1 (Figure 5b). A characteristic tumour cell pattern of membranous and cytoplasmic staining was visible, ranging from weak to strong intensity (Figure 5b, insets for T2 and R1). In general, tumour cells at the invasive margin stained strongest for PD-L1 expression compared to the tumour cells in the centre. Indeed, most tumour cells in the centre did not express PD-L1. In T1 and T3, PD-L1 expression was observed on tumour-associated immune cells only and not on the tumour cells (Figure 5b). Of notice, in the T3 sample, background staining was visible in the mucosal tissue. As shown by RNA-seq, IHC data confirmed the strong immunosuppressive environment in T2 and R1.

The results reported here describe the intratumoural immune profile by RNA-seq and histological analyses, showing that this patient was carrying highly immunosuppressive tumours.

## 4. Discussion

In this ontological study, we used whole-exome and RNA sequencing to examine heterogeneity in the genetic profiles of three synchronous primary cancers and one recurrence to describe how tumour heterogeneity within an individual patient confers different molecular and cellular properties that may have ultimately influenced the clinical characteristics and treatment response observed in the patient.

In the clinical setting, H&E staining and additional immunostaining markers (e.g., TP40, TP53, MKI67, and KRT5) are frequently used to assess whether the recurrent tumour shares any histopathological features with the primary tumour. It would be very difficult, if not impossible, however, to evaluate the differences between the three primary tumours and any relation to the recurrence in this particular case using IHC. Nevertheless, using WES we could illustrate that the three tumours were genetically very different: only two mutations were found to be shared between primary tumours T1 and T3, and none of their mutations were shared with primary tumour T2. This heterogeneity was also reflected by the low correlation among the primary tumour samples in the analysis of copy number profiles. Moreover, we could show that 120 mutations of the recurrent tumour R1 were only shared with T2 and not with either of the other primaries, demonstrating that the recurrence originated from this tumour. This was further supported by the results of the copy number analysis, where the R1 profile was much closer to that of T2 than to T1 and T3. These findings indicate that WES is very useful in uncovering the origin of the recurrent tumour in the case of multiple primary tumours. Notably, we identified a mutation shared by T2 and R1 in ATM, a gene that is implicated in chemotherapy resistance [41] and which may have conferred this phenotype to the recurrent tumour. The patient did not, in fact, respond to first-line platinum-based chemotherapy [41]. Although it is inconclusive whether this mutation was causative to resistance, our data indicate how such detailed genetic information may change the management plan for patients affected by several primary tumours, especially those from different anatomical sites, e.g., SCC of tongue and lung. This is further underlined by the finding that R1 acquired additional mutations that, according to their signatures, are at least partially the result of the treatment itself.

Our results are consistent with two studies describing the genomic analysis of multiple synchronous lung and colorectal cancers [42,43]. These two studies showed that synchronous primaries in the same patients had distinct genomic profiles, which were driven by different molecular events and were no more similar to each other than tumours from different patients [42,43]. In another case report, a divergent EGFR gene profile was found in a patient who presented with multiple synchronous tumour lesions in separate lungs [44], although only a limited gene panel was used in this study, and certain actionable genes might have been missed with this approach. By utilising a limited gene panel testing, such as the FDA-approved FoundationOne CDx from Foundation Medicine, we would have detected four sSNVs common to T2 and R1 and no sSNV shared between R1 and either T1 or T3: although this result would have suggested the origin of the recurrence, WES analysis afforded an unambiguous and definitive assessment.

The analysis of the patient’s germline landscape revealed several variants in genes known to be associated with familial cancers, an observation that could partly explain why this patient developed multiple synchronous primaries. Furthermore, several genes affected by germline variants are associated with DNA damage sensing and double-strand repair, potentially conferring a predisposition for a higher mutation rate, which in turn might have driven the development of three independent tumours. This is also supported by the presence of mutational signatures 20 and 6 in T1 and T3, respectively, both of which have been associated with defective DNA mismatch repair [38]. Furthermore, the high number of individual somatic mutations in genes associated with tumour suppression indicates that these were acquired independently, giving rise to three primary tumours with a very different genetic and molecular makeup, as well as a heterogeneous gene expression landscape. Other germline defects found in genes of the TP53 pathway and in genes involved in immune function and tumour immune surveillance may also have contributed to carcinogenesis. Additionally, the long-term tobacco and alcohol exposure was also likely a major risk factor, reflected by the consistent occurrence of Signature 16 among all tumours, which represents the biggest fraction of somatic mutations (Figure 1c).

From a therapeutic point of view, the germline variants found in genes involved in DNA damage response and double-strand repair mechanisms indicate the tumours could have been sensitive to the treatment with PARP1/2 inhibitors such as olaparib and niraparib through mechanisms of synthetic lethality [45]. These could have represented a potential therapeutic option for this patient. In addition, all the tumours had a high tumour mutational burden, indicating microsatellite instability and that the patient may have responded well to checkpoint blockage therapies [46]. Checkpoint blockade as a promising treatment option was also suggested by the higher expression levels of PD-L1, especially in T2 and R1.

In order to further investigate the immune microenvironment and better understand its contribution to tumour development and relapse, we applied the quanTIseq deconvolution pipeline [22] to quantify the immune cell subsets infiltrated into the tumour samples. Among the three primary tumours, the microenvironment in T2 showed particularly unfavourable characteristics, such as the high frequency of regulatory T cells, M2 macrophages and neutrophils (Figure 4a). In combination with the high expression levels of PD-L1 on tumour cells and of TIM-3 and LAG-3, which are typical markers of exhausted T cells (Figure 5), taken together, this evidence suggests that T2 had a highly immunosuppressive environment. These conditions likely contributed to the tumour immune escape [47] and favoured the relapse, which occurred 14 months after primary resection.

Based on the quanTIseq analysis, T3 could have had a higher chance of responding to immunotherapy than T1 and T2, in view of higher M1/M2 macrophages, lower frequency of regulatory T cells, detectable percentages of dendritic cells and CD4+ T cells, in addition to a high tumour mutational burden and defective DNA mismatch repair signature [46,48]. The very low frequency of CD8+ T cells in this tumour, however, did not favour a good prognosis.

This case shows that immunosuppression exerted by regulatory T cells, M2 macrophages, and neutrophils may have been present in all primary tumours, and would have determined the failure of an obvious anti-tumour immune response generally conferred by cytotoxic T cells. A deeper understanding of a potential role, for example, of myeloid-derived suppressor cells (MDSCs) as a subset of neutrophils in immunosuppression would require further bioinformatics evaluation.

Interestingly, R1 had a high macrophage M1/M2 ratio, diverging from its origin T2. This shift of macrophage phenotype towards M1 polarization could be due to the chemotherapy received by the patient, a phenomenon already described by others [49,50]. Overall, these immunological observations would thus support the notion that immunotherapy treatment following chemotherapy could be beneficial for HNSCC patients. On the other hand, R1 had reduced proportions of CD8+ T cells and B cells compared to T2, supporting again the hypothesis that immune escape might have played a role in the recurrence of this patient.

In the era of cancer multi-omics, our study shows that a comprehensive genomic and transcriptomic analysis can yield important clinical value, such as contributing to treatment plan revision for some patients with synchronous primaries and matched recurrent tumours. In a case report of a patient with lethal metastatic prostate cancer, the genomic analyses of primary cancer and seven matched metachronous metastases showed that the lethal clone arose from a small focus of low-grade cancer in the primary tumour, but not from the bulk of higher-grade primary cancer [51]. Our data are in line with previous patient reports [51,52] and underlines the value of dissecting tumour heterogeneity, mapping tumour progression, and evolution within individuals: such a deeper understanding of cancer onset and progression is key to refining personalised immunotherapies. Building a common database of individual cases would ease the cross-comparison among individuals and help identify recurring patterns that would advance more robust conclusions on the diagnosis and progression of synchronous and metachronous tumours [52]. The data from this particular case specifically highlights how the individual genetic profile of each tumour drives a clearer understanding of clinical characteristics and response, and in turn, infers individual treatment efficacy and options.

## Figures and Tables

**Table 1:** Clinical, sequencing and histological profile of samples investigated in this study. Sample type, number of sSNVs (total number and non-synonymous sSNVs in parentheses) detected in the WES data, tumour purity estimated by the mutation detection pipeline, tumour area, and tumour nuclei from the histological inspection are shown for each sample.

Sample	Type	All sSNVs (Non-Synonymous)	Purity	Tumour Area (%)	Tumour Nuclei (%)
Primary T1	Biopsy	143 (101)	0.18	20	35
Primary T2	Biopsy	125 (78)	0.16	40	40
Primary T3	Biopsy	122 (74)	0.68	80	80
Recurrence R1	Biopsy	217 (147)	≤0.64	90	90

## Data Availability

All sequencing data that support the findings of this study have been deposited at the European Genome-Phenome Archive (http://www.ebi.ac.uk/ega/ (accessed on 10 March 2021)), which is hosted by the European Bioinformatics Institute, and are accessible through the accession number EGAS00001004857.

## Supplementary Materials

The following are available online at https://www.mdpi.com/article/10.3390/ijms22147583/s1.
