# Supplementary figures and images for: Comprehensive Genomic and Transcriptomic Analysis of Three Synchronous Primary Tumours and a Recurrence from a Head and Neck Cancer Patient
Source: Int J Mol Sci. 2021 Jul 15;22(14):7583. doi: 10.3390/ijms22147583 (PMC8305204; doi:10.3390/ijms22147583)

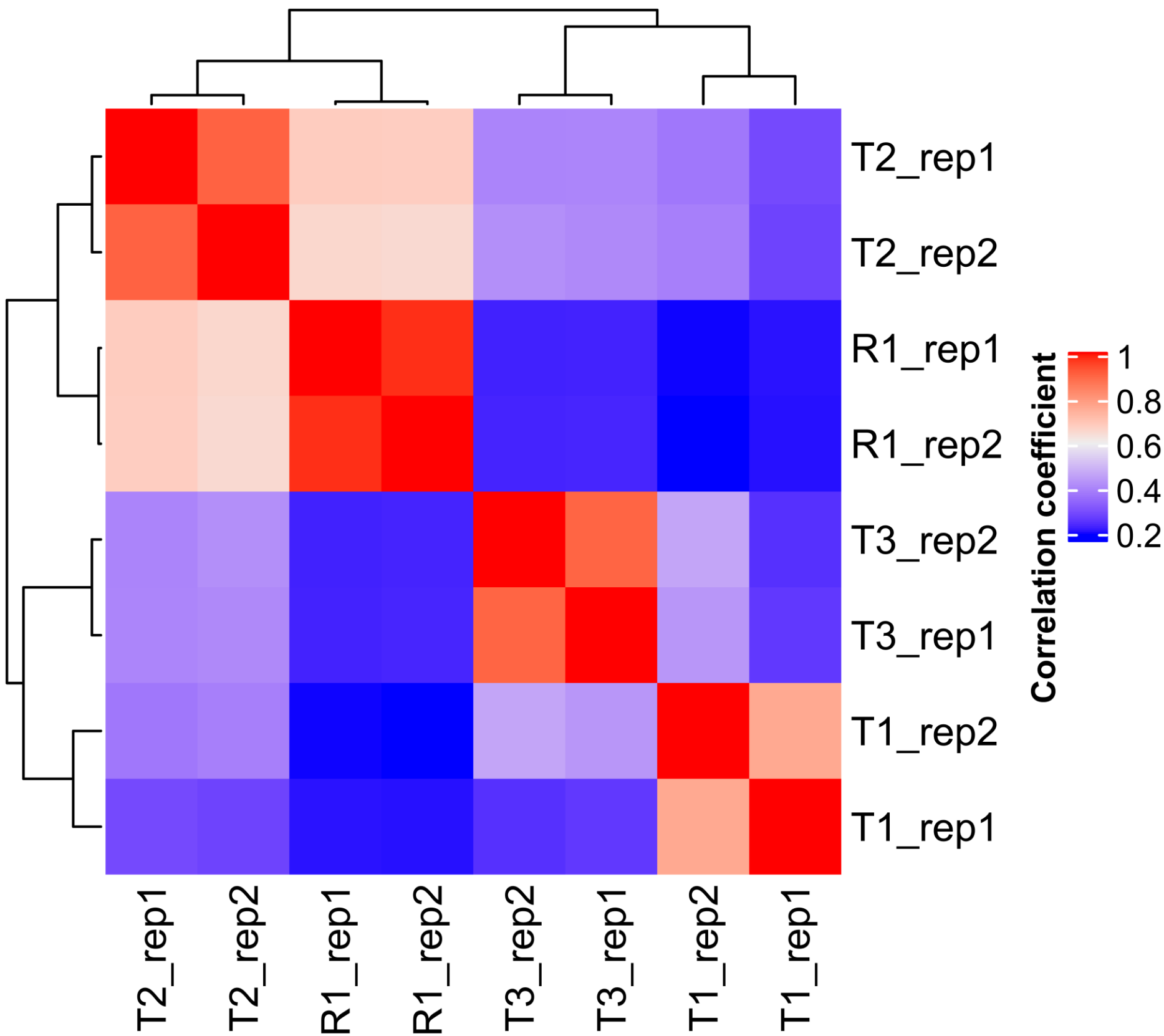

Supplement: Supplementary file 1 [file ijms-22-07583-s001.zip › SupplementaryFig_1.pdf]

CD3 IHC

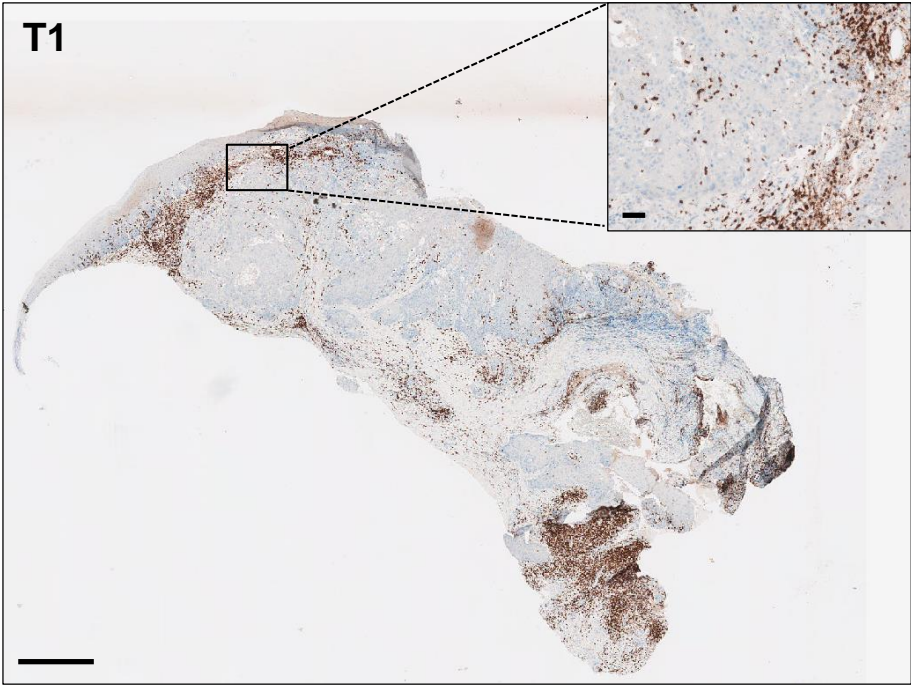

CD3 IHC

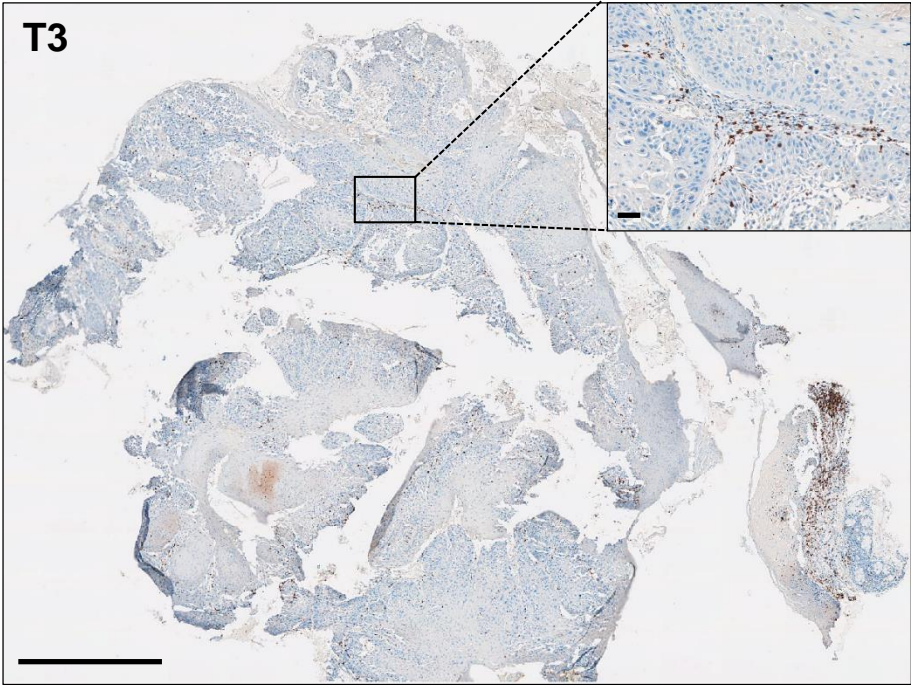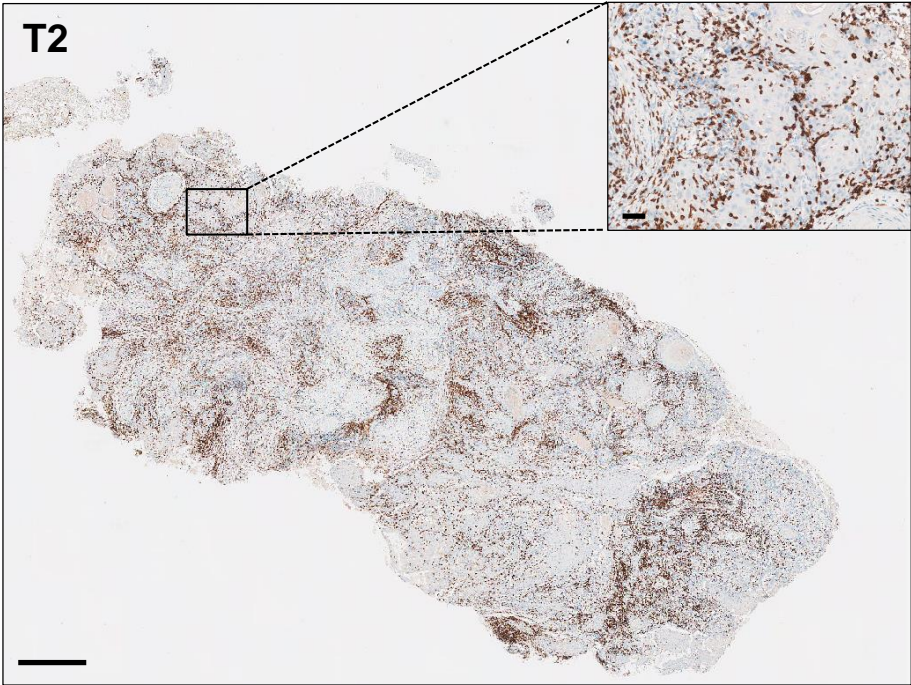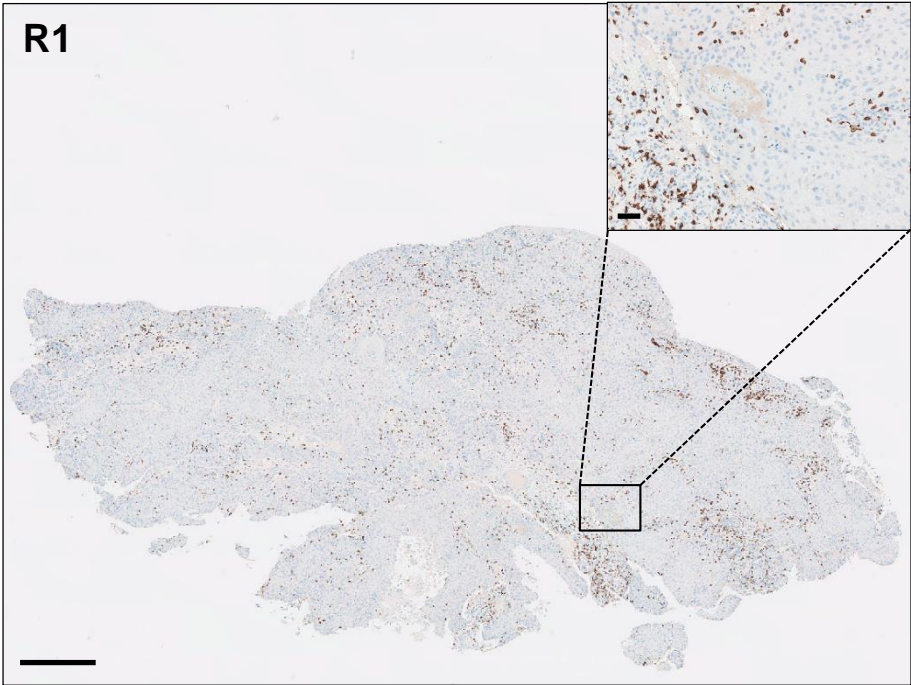

Supplement: Supplementary file 1 [file ijms-22-07583-s001.zip › SupplementaryFig_2.pdf]

CD163/CD68 mIHC

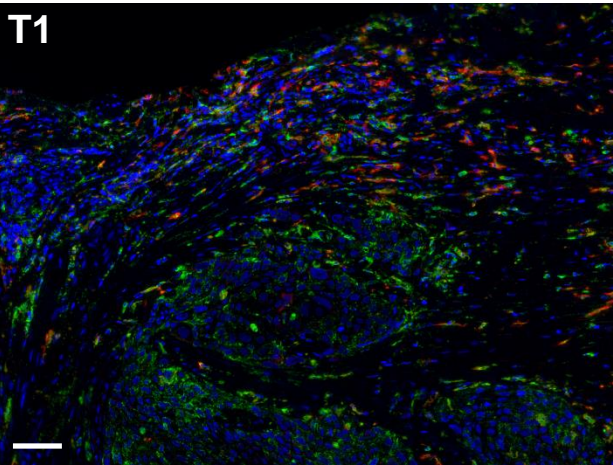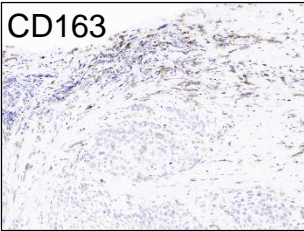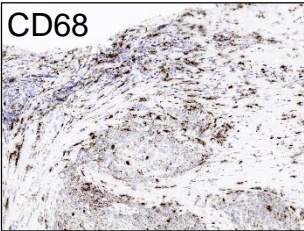

CD163/CD68 mIHC

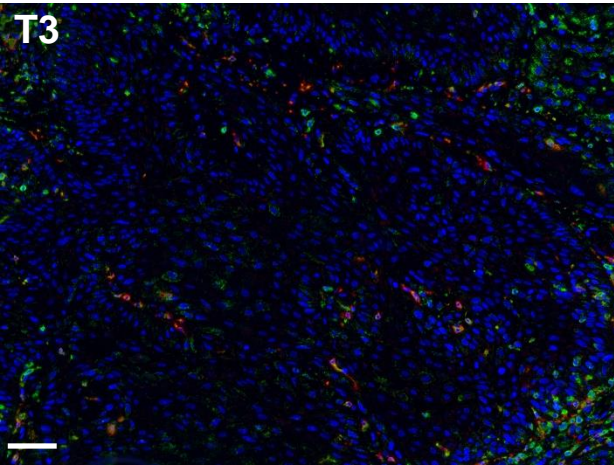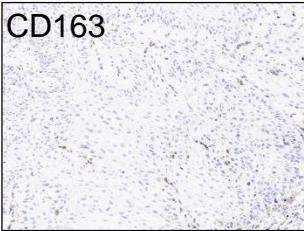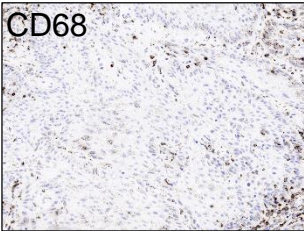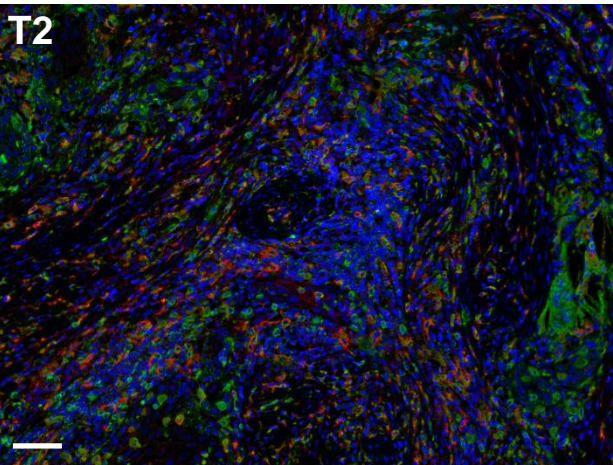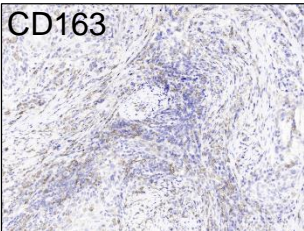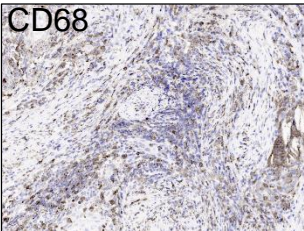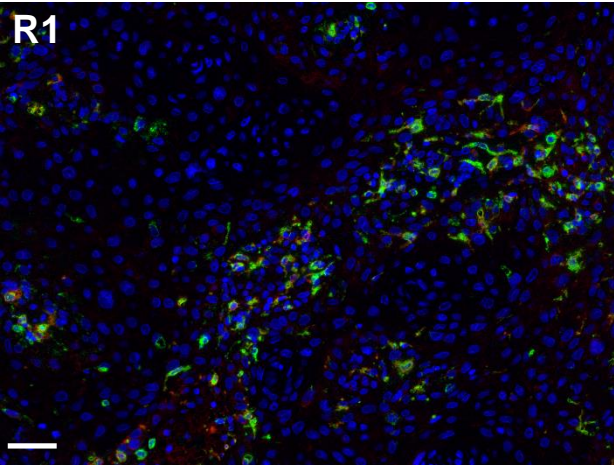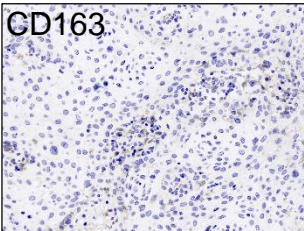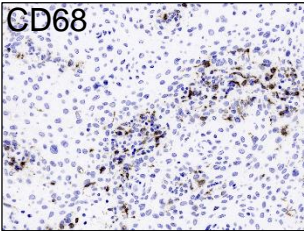

Supplement: Supplementary file 1 [file ijms-22-07583-s001.zip › SupplementaryFig_3.pdf]
